# CMOS-Based Gas Direction Sensors with a Surface-Integrated Pillar

**DOI:** 10.3390/s26082364

**Published:** 2026-04-11

**Authors:** Yusuke Yodo, Kazunari Lucas Cerizza Freitas, Yoshihiro Asada, Toshihiko Noda, Kazuaki Sawada, Masahiro Akiyama

**Affiliations:** 1 National Institute of Technology (KOSEN), Nagano College, Nagano 381-8550, Japan; y_sato@nagano-nct.ac.jp (Y.Y.); cerizza.freitas.lucas.kazunari.hx@tut.jp (K.L.C.F.); 2 Department of Electrical and Electronic Information Engineering, Faculty of Engineering, Toyohashi University of Technology, Toyohashi 441-8580, Japan; asada.yoshihiro.vs@tut.jp; 3 Institute for Research on Next-Generation Semiconductor and Sensing Science, Toyohashi University of Technology, Toyohashi 441-8580, Japan; noda.toshihiko.zk@tut.jp (T.N.); kazuaki.sawada@tut.jp (K.S.)

**Keywords:** CMOS gas sensor, CMOS potential sensor, gas direction estimation, gas source localization, sensor array, single-chip sensor

## Abstract

Conventional gas direction estimation methods that rely on concentration gradients or time-of-arrival differences typically require multiple spatially dispersed sensors, leading to increased system bulkiness and complexity. Furthermore, previous CMOS-based approaches that relied on gas diffusion struggled to achieve stable direction estimation in high-speed airflow environments. To address these challenges, we propose a streamlined method integrating a pillar onto a single CMOS gas-sensor array, eliminating additional MEMS fabrication. This approach exploits a fluid dynamic phenomenon where the pillar creates a distinct flow “shadow” pattern (a region of localized gas dilution) on the sensor surface. Experimental verification using ammonia gas confirmed that this “shadow” is clearly observable as a localized reduction in sensor output under high-speed turbulent flow. Crucially, the spatial position of this pattern correlates strongly with the direction of gas inflow. This study demonstrates the feasibility of gas direction estimation using a single chip, paving the way for high-precision detection in challenging, rapid-airflow environments.

## 1. Introduction

Technologies for detecting trace amounts of specific chemical substances, such as landmine detection, gas leak identification, and early fire detection, play an extremely crucial role in crisis management. Particularly in environments with airflow, such as natural settings, reliable direction detection still relies on the capabilities of biological organisms (e.g., rats for landmine detection and drug sniffer dogs) for certain applications [1,2,3].

Recently, fundamental technologies and sensing materials for MEMS gas sensors have remarkably advanced. Building upon these hardware improvements, sophisticated gas sensing systems—integrated with machine learning algorithms, self-calibration strategies, and miniaturized electronic noses—are increasingly demanded for intelligent IoT monitoring and robotics applications. In these highly complex environments, distinguishing the spatial direction of odor sources or gas leakages remains a critical yet challenging capability [4,5,6,7].

In the field of gas-source localization, two main approaches have been explored. One is the “Concentration Gradient Method,” which utilizes the difference in gas concentration detected by multiple sensors. The other is the “Time-of-Arrival Difference Method,” which measures the time difference until the gas reaches each sensor. However, both the conventional methods have fundamental limitations. The Concentration Gradient Method is susceptible to signal complexity and instability in turbulent environments, whereas the Time-of-Arrival Difference Method requires a physical distance between the sensors to detect a clear time difference, imposing a constraint that means the system tends to become bulky and complex [8,9].

The basic technology for gas-source localization was established by Ishida et al. [10,11] in the 1990s; however, their approach encountered the problem of system bulkiness. Furthermore, the semiconductor oxide gas sensors require operation at high temperature (200–450 °C), resulting in high power consumption and the severe problem of thermal convection caused by the sensor itself, which introduces measurement errors [12,13,14].

Recently, gas sensors that respond to specific chemical substances have been developed by applying room-temperature operation with a low-power CMOS potential sensor array device coated with sensitive membranes in the sensing area (SA) [15,16,17,18]. Because these CMOS sensors do not require high-temperature operation, they eliminate the risk of thermal convection caused by the sensor. Our group previously leveraged this advantage by proposing and experimentally verifying methods for direction estimation using CMOS sensor arrays based on the gas diffusion speed and concentration gradients [17,18,19]. However, these approaches inherently rely on microscopic and unstable information regarding gas diffusion and concentration gradients. Consequently, stable direction estimation remains difficult in high-speed airflow environments such as outdoor settings or ventilation systems, where turbulence readily disrupts the concentration gradient [20,21].

To overcome the limitations of conventional studies, we propose a novel principle: installing pillars on the CMOS gas sensor array to utilize the flow “shadow” created by the gas stream. This approach leverages fluid dynamics to capture stable reaction patterns directly on a single chip, thereby enabling robust direction estimation even in high-speed airflow environments without requiring spatially dispersed sensors.

## 2. Materials and Methods

### 2.1. Experimental Setup and Gas Injection

In this experiment, a 256 × 256 pixel CMOS gas sensor array capable of two-dimensional potential imaging was employed. The detailed circuit architecture and operating principles utilizing the charge-transfer technique are described in Section 2.3.

**Figure 1:**
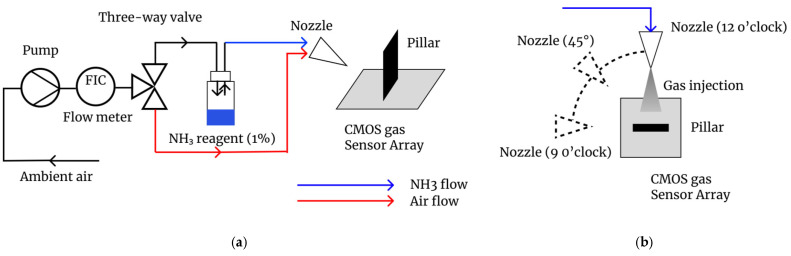
Schematic diagram of the experimental setup for gas sensing and directional flow testing. (**a**) Overview of the gas delivery system, including the pump, flow meter, and a three-way valve used to switch between blank air and the target NH_3_ gas. (**b**) Illustration of the localized gas injection setup, showing the three different nozzle angles (vertical at 12 o’clock, diagonal at 45°, and lateral at 9 o’clock) relative to the pillar and the CMOS gas sensor array.

Experiments were conducted using NH_3_ gas to verify the sensor performance. Air was pumped into a bottle containing 1% NH_3_ reagent (diluted from a 10% ammonia solution; FUJIFILM Wako Pure Chemical Corporation, Osaka, Japan), and a three-way valve was used to switch between the target gas and blank air (Figure 1a). The measurement cycle was set to 300 s: air injection (0–180 s), NH_3_ gas injection (180–240 s), and air purging (240–300 s). The gas flow rate was monitored at 500 mL/min using a variable-area flow meter (NFM-V-S-A-5; Techne Keisoku Co., Ltd., Kawasaki, Japan), and the gas was delivered by a linear vacuum pump (LV-125A; Nitto Kohki Co., Ltd., Tokyo, Japan). To clarify the gas-sensing measurement setup and airflow testing platform, a detailed schematic diagram of the experimental system is provided. All measurements were performed at room temperature and atmospheric pressure in a still-air (windless) environment to eliminate external airflow interference.

**Figure 2:**
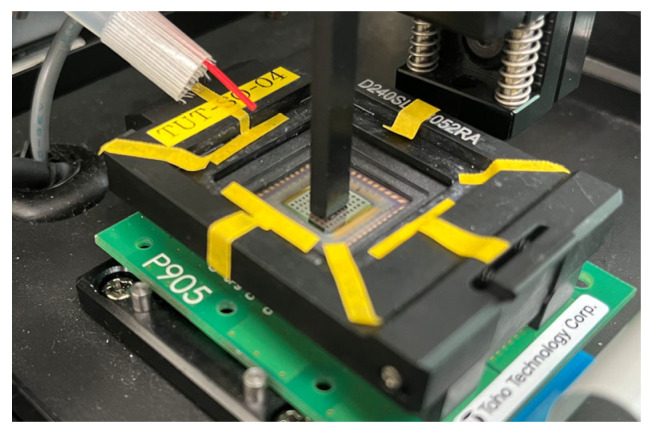
An example of the structure and fixation method for the wall-shaped pillar installation on the sensor surface.

A separate wall-shaped pillar made of conductive rubber (width: 5 mm; thickness: 1 mm) was placed directly onto the sensor surface (Figure 2). The conductive rubber material was selected to avoid affecting the sensor potential. This simple installation method allows for the use of existing CMOS gas sensor chips without any additional micro-fabrication processes. The pillar was mechanically fixed using a clamp from the top. To verify the effect of the structure, we compared sensor responses with and without the pillar. Additionally, to test the directional sensitivity, gas was supplied from three different angles: vertical (12 o’clock), diagonal (45° from the top left), and side (9 o’clock) as illustrated in Figure 1b.

### 2.2. Gas Direction Estimation Method Using CMOS Gas Sensor

In this study, to overcome the limitations of conventional methods, we propose a novel gas direction estimation method that utilizes a characteristic reaction pattern (referred to as a “shadow”) formed on the sensor by the gas flow, achieved by installing a specific structure on the reactive area of the CMOS gas sensor.

**Figure 3:**
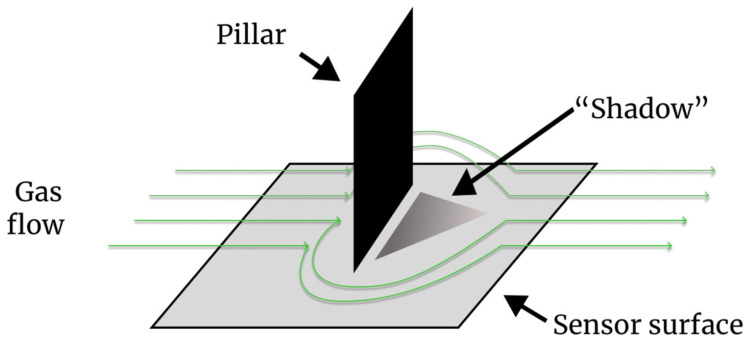
Conceptual diagram of the proposed principle: formation of a gas concentration “shadow” behind the pillar.

The main idea of the proposed method is to introduce pillar-like structures (pillars), onto the surface of a CMOS gas sensor. The principle behind the introduction of these pillars was based on fluid dynamics. When gas flows around the side of a pillar, a wake region is formed behind it. In this wake region, the flow velocity decreases, and the gas concentration is locally diluted. This diluted region is observed as a characteristic pattern, or “shadow,” on the output of the CMOS gas sensor. Figure 3 shows a conceptual diagram of the proposed principle.

The concept behind the proposed method is extremely simple. The direction of the formed “shadow” directly indicates the flow direction of the gas incident on the sensor. Specifically, because this “shadow” is always formed on the side opposite to the direction from which the gas is blown, capturing the position and characteristics of this “shadow” makes it possible to estimate the direction of the gas source instantly and without the need for distance differences between sensors. A key advantage of this method is its simplicity and compatibility. Since the “shadow” is generated by a macroscopic physical obstacle, the pillar does not need to be monolithically integrated into the sensor chip using complex MEMS processes. Instead, a separate structure can simply be placed onto an existing, commercially available CMOS gas sensor array. This allows for a low-cost and flexible system configuration.

To clarify the structural and design advantages of the proposed method, a qualitative and semi-quantitative comparison with conventional gas direction estimation techniques is summarized in Table 1. The conventional concentration gradient and time-of-arrival difference methods fundamentally require multiple spatially dispersed sensors to detect macroscopic differences, which inevitably increases the system’s physical dimensions and complexity. In contrast, the proposed method leverages a single high-density CMOS sensor array combined with a passive surface pillar. By intentionally generating a localized flow “shadow,” it extracts directional information directly from the fluid dynamic interaction on a single chip. This single-chip architecture drastically reduces spatial constraints and required sensor count while maintaining robust direction estimation principles suitable for high-speed airflow environments.

### 2.3. Characteristics and Operating Principle of the CMOS Gas Sensor

The CMOS gas sensor used in this study is based on the charge-transfer-type CMOS image sensor technology developed by Toyohashi University of Technology [15,16]. This technology converts minute potential changes into charge quantities and utilizes a charge transfer technique to visualize the magnitude of potential changes. This technology has been successfully used to image hydrogen ion (pH) distributions (proton imaging) [17,18]. Notably, the densification of this image sensor progressed, achieving a sensor with a 1024 × 1024 pixel configuration and a high-density pixel size of 2.1 × 2.1 µm [16].

**Figure 4:**
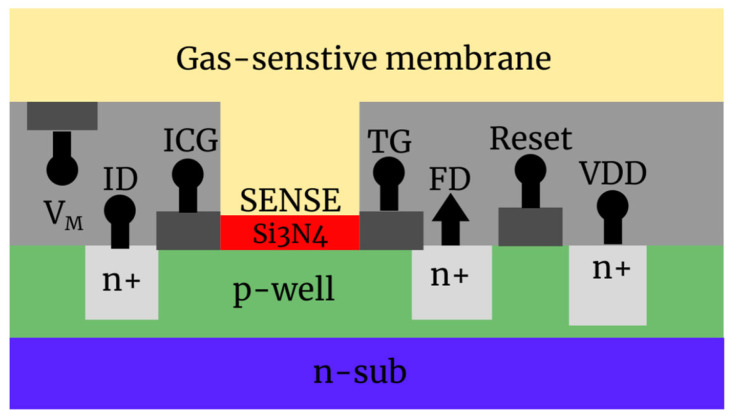
Pixel structure and main components of the CMOS gas sensor.

The CMOS gas sensor employed in this study was based on the same type of CMOS potential sensor array used in previous studies that utilized this technology. It was constructed by uniformly coating a gas-sensitive membrane (an organic semiconductor thin film such as polyaniline (PANI)) onto the SA of the potential sensor array [19,22]. Membrane bias ($V_{M}$) was applied to the coated membrane to determine the potential of the sensor array (Figure 4).

The pixel structure of a CMOS sensor array (Figure 4) comprises the basic components of a charge-transfer-type CMOS image sensor. The sensor was fabricated on a p-type well (p-well) on an n-type semiconductor substrate and detected potential changes in the odor-sensitive membrane through the following key elements:

- SENSE (Sensor Area): In this study, this functioned as the sensing part that detected changes in the membrane charge due to gas adsorption as potential changes via the PANI-coated gas-sensitive membrane;
- Floating Diffusion (FD): A charge detection node composed of an n+ region. The charge accumulated in the SENSE region is transferred to the FD node and read out as a potential change (signal) corresponding to the amount of charge;
- Transfer Gate (TG): Gate-control electrode for transferring the charge accumulated in the SENSE layer to the FD;
- Input Diode (ID)/n+ region: A p-n junction that supplies charge to the SENSE region. Nodes that handle charges such as FD and ID are composed of n-type highly doped regions (n+) formed within the p-well;
- VDD: Power supply voltage, supplying power for the operation of the entire sensor chip;
- Reset: Control signal to initialize the potential of the FD node. It resets the potential of the FD, which is the charge detection node, returning it to its state before the start of the measurement. This reset operation is typically performed for each frame or readout cycle to ensure accurate signal measurement;
- Input Control Gate (ICG): An electrode for gating and controlling the potential levels. It controls the FD so that it can only detect the selected sensor region. This functions as a reference for controlling the potential level of the SENSE region during the charge-filling process. Additionally, it sets the reference potential for converting potential changes due to gas adsorption into charge via the “fill and spill” technique;
- $V_{M}$: A constant voltage is applied to the sensitive membrane to fix the potential of the SA

**Figure 5:**
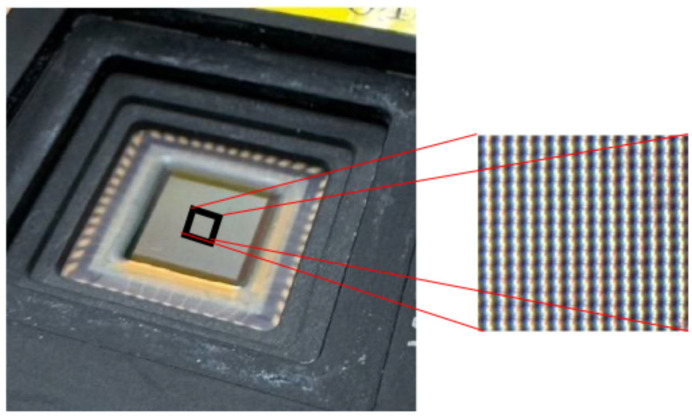
Overall configuration of the high-density CMOS gas sensor array.

This sensor is configured as a high-density 256 × 256 pixel two-dimensional sensor array, with each pixel arranged at a pitch of 30 µm. This realizes a detection area of several square millimeters, and because a large number of sensor units are densely integrated, it functions as a sensor array capable of detecting gas/odor and estimating direction over a wide range (Figure 5).

The operating principle of a CMOS gas sensor is based on the fact that when a sensitive membrane on the sensor surface adsorbs specific odor/gas molecules, the charge of the membrane changes, resulting in a change in the potential of the sensor surface. By detecting the change in the amount of charge in the sensor region (SENSE) caused by this potential change, the sensor can detect gases by measuring the degree of potential change before and after odor adsorption. This CMOS gas sensor has advantages over the widely used semiconductor gas sensors (MOS sensors). While conventional semiconductor gas sensors require operation at high temperatures of 200–400 °C and the heat generation causes thermal convection that disturbs weak gas flows—a fundamental source of error—this CMOS sensor can operate at room temperature and carries no risk of air convection caused by a heater. Furthermore, CMOS technology offers the advantages of easy integration and multiplexing.

**Figure 6:**
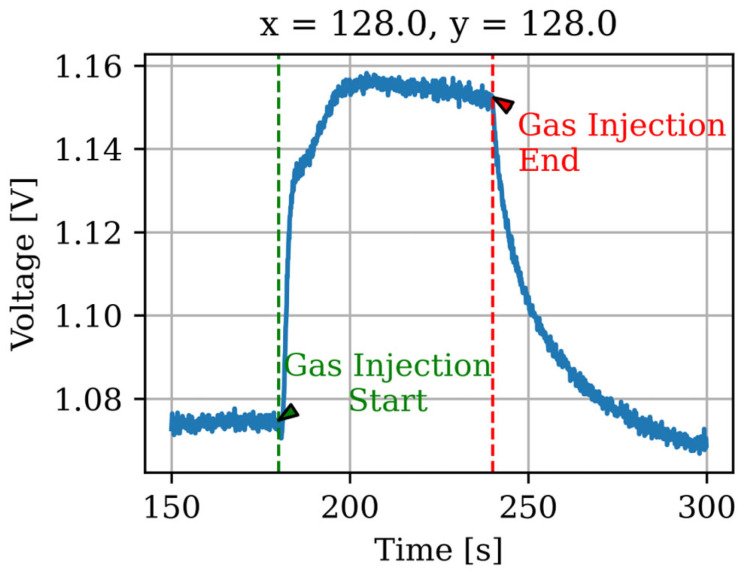
Time response of sensor output potential during ammonia gas injection.

Figure 6 shows the specific behavior of one point in the sensor array when measuring ammonia gas, illustrating how the potential changes over time. The vertical axis represents the potential, and the horizontal axis represents the elapsed time. Ammonia gas was injected at 170 s and was completed after 240 s. The reaction speed, intensity, and decay rate after the injection depend on the sensitive membrane, target substance, concentration, and coating method. Previous studies formed membranes at regular intervals on the sensor array, whereas the sensor array used in this study was characterized by a uniform coating of the sensitive membrane such that the behavior of each sensor was identical.

By exploiting the advantages of this CMOS sensor array, our group proposed a method for estimating gas direction using integrated CMOS odor sensors. Previously, Asada et al. [19] proposed and experimentally verified two methods for estimating the direction of a gas source using a CMOS gas sensor; the same CMOS potential sensor array was used for this study with a sensitive membrane coating. One direction estimation method utilizes the gas diffusion speed, where membranes closer to the gas source respond sequentially. The other method utilizes the concentration gradient, where the concentration increases closer to the gas source. Although these conventional methods benefit from the advantages of CMOS sensors (i.e., elimination of thermal convection), they inherently rely on microscopic and unstable information such as the concentration gradient caused by gas diffusion. Therefore, in situations where high-speed flow is dominant, such as in outdoor environments or ventilation systems, turbulence readily disrupts the concentration gradient, posing the challenge of making stable direction estimation difficult.

### 2.4. Fluid Dynamic Analysis of Wake Generation

To verify the formation of a wake—specifically Karman vortex streets [23]—behind the pillar, we evaluated the flow field around the structure. The Reynolds number (*Re*) was calculated to characterize the flow regime. For the prismatic pillar with a characteristic length equal to the pillar width (L=0.005 m) and a representative velocity at the pillar (v ≈10 m/s), *Re* is expressed as:

(1) $$Re=\frac{\rho vL}{\mu },$$

The representative velocity v was estimated based on axisymmetric free jet decay theory [24,25]. Because the distance from the nozzle exit to the pillar (approximately 30 mm) far exceeds the potential core length (typically 5d to 6d, where d = 0.3–0.5 mm is the nozzle diameter), the jet has fully entered the self-similar decay region at the pillar location, where the centerline velocity attenuates to approximately one-tenth of the nozzle exit velocity. Using an air density of ρ=1.225 kg/m^3^ and a dynamic viscosity of $\mu =1.81\times 10^{-5}$ Pa·s, the calculated *Re* is approximately 3380. This value far exceeds the threshold for regular Kármán vortex shedding (*Re* ≈ 40–300), confirming fully turbulent wake conditions where shear-layer instabilities promote mixing and local pressure reduction [26]. In such wakes, reduced flow velocity and vortex structures are known to cause local gas concentration dilution and inhomogeneity [27,28,29], which we utilize as the “shadow” pattern for direction estimation.

Furthermore, to evaluate the dominance of advection over diffusion, we calculated the Péclet number (Pe):

(2) $$Pe=\frac{vL}{D},$$

Using the gas diffusion coefficient D for NH_3_ in air (approx. $0.28\times 10^{-4}$ m^2^/s), the result is Pe≈1780≫1. This formally demonstrates that advection overwhelmingly dominates diffusion, providing a strong theoretical basis that the localized gas dilution (“Flow shadow”) will be firmly sustained without being disrupted by rapid gas diffusion into the wake.

## 3. Results

### 3.1. Comparison of Output with and Without Pillar

**Figure 7:**
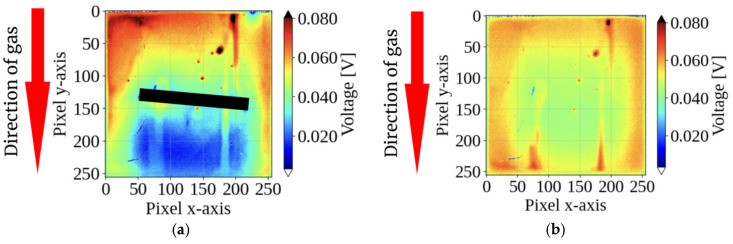
Comparison of sensor response patterns under vertical gas flow (from 12 o’clock): (**a**) with the pillar and (**b**) without the pillar. The integrated pillar in (**a**) structurally obstructs the direct airflow, creating a distinct “flow shadow” with 30–50% signal attenuation on the downstream side, whereas the sensor without a pillar in (**b**) shows no such selective pattern. The images represent the sensor response at 2 s after gas injection.

Figure 7 compares the sensor response to vertical gas inflow (12 o’clock). With the integrated pillar, a distinct “shadow” region with locally low potential formed on the downstream side, exhibiting signal attenuation of 30–50% compared to the surrounding area. In contrast, the sensor without a pillar showed no clear pattern under identical flow conditions. These results experimentally validate the proposed principle of gas direction detection via structurally generated shadows.

### 3.2. Response at Different Angles

**Figure 8:**
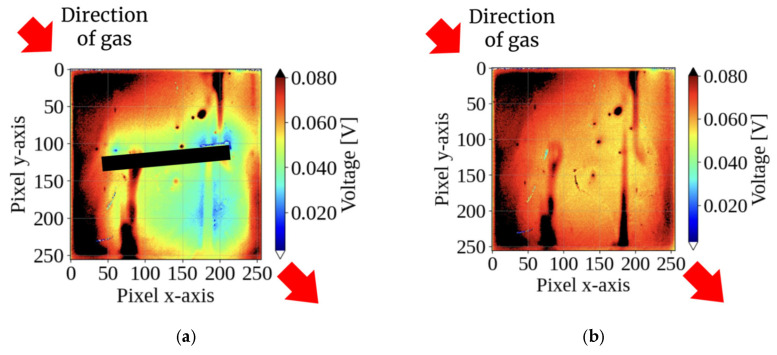
Output potential patterns under diagonal gas flow (from 45°, top left): (**a**) with the pillar and (**b**) without the pillar. Even under a non-frontal flow angle, the pillar in (**a**) successfully generates a recognizable shadow region on the opposite side (bottom right), demonstrating the robust directional selectivity of the proposed structure compared to the uniform response in (**b**). The images represent the sensor response at 2 s after gas injection.

The directional selectivity of the sensor was further tested. For diagonal gas inflow (45° from the top left), a clear “shadow” formed on the bottom right, directly opposite the inflow direction (Figure 8). This confirms that the proposed method maintains reliable directional selectivity for non-frontal flows.

### 3.3. Response to Side Inflow

**Figure 9:**
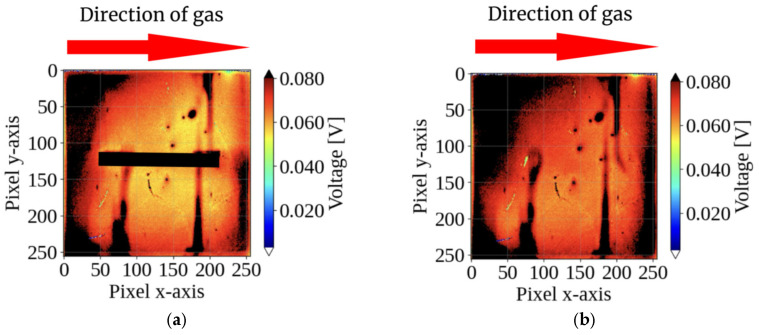
Sensor response under side gas flow (from 9 o’clock): (**a**) with the pillar and (**b**) without the pillar. In this case, where the gas flows parallel to the rectangular pillar, the shadow formation is absent in both (**a**) and (**b**). This effectively indicates that the current pillar geometry is specifically optimized for frontal and diagonal flows. The images represent the sensor response at 2 s after gas injection.

For side inflow (9 o’clock), however, no distinct shadow was observed (Figure 9). This lack of response is attributed to the current wall-shaped pillar geometry, which is optimized primarily for frontal and near-frontal flows. Structural optimization to achieve omnidirectional sensitivity is discussed in the subsequent sections.

## 4. Discussion

### 4.1. Effectiveness and Physical Interpretation of the Proposed Method

This study demonstrates a method for visualizing the gas direction using pillars on a CMOS sensor array. Figure 7 and Figure 8 show that the interaction between the gas flow and the structure creates a distinct “shadow” on the downstream side.

As our experimental model represents an idealized condition (a steady source in motionless air), it serves as a crucial Proof of Concept (PoC). This controlled environment is essential for quantitatively isolating and verifying the underlying fluid-dynamic mechanism without interference from unpredictable environmental noise. The mechanism of “shadow” formation is fully consistent with the fluid dynamics analysis presented in Section 2.4. Under the evaluated turbulent wake conditions, reduced flow velocity and suppressed mixing lead to localized dilution of the gas concentration.

Furthermore, the interaction mechanisms responsible for the sensor’s selectivity toward the target gas, specifically between PANI and NH_3_ (including hydrogen bonding and dedoping mechanisms), have been well established by recent theoretical analyses based on computational methods such as Density Functional Theory (DFT) [30] and Molecular Dynamics (MD) simulations [31]. Therefore, our sensor reliably exploits these proven interactions.

**Figure 10:**
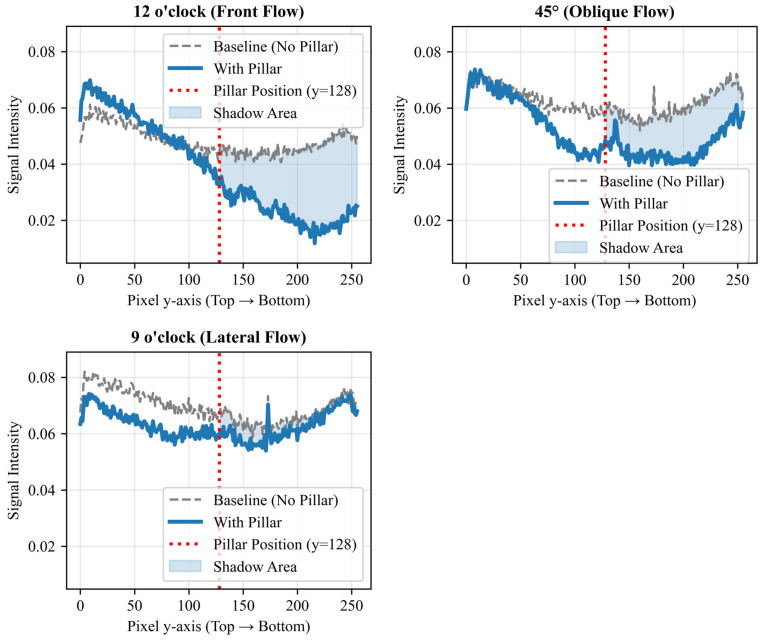
Extracted 1D vertical cross-sectional profiles of the sensor output at x = 128 under various gas inflow directions (12 o’clock, 45°, and 9 o’clock) compared to the baseline condition. The downstream region (y ≥128) clearly exhibits localized signal attenuation (“flow shadow”), with the magnitude of the drop demonstrating strong angular dependence.

To further substantiate the proposed direction-estimation principle, we quantitatively analyzed the “flow shadow” phenomenon by extracting 1D vertical cross-sectional profiles at a fixed spatial coordinate (x=128) for each inflow condition. To visually demonstrate this flow shadow phenomenon, the extracted 1D cross-sectional profiles for the baseline and the three inflow conditions are plotted in Figure 10. To ensure a standardized evaluation across all flow angles, the sensor surface was divided into an upper half (y < 128) and a lower (shadow) half (y ≥128). Based on these profiles, we calculated three distinct metrics:

1. Upper vs. Lower Difference ($\Delta S_{\mathrm{diff}}$): The signal drop within the single image.
  (3) $$\Delta S_{\mathrm{diff}}=\left(\frac{1}{128}\sum_{y=0}^{127}S_{\mathrm{pillar}}\left(128,y\right)\right)-\left(\frac{1}{128}\sum_{y=128}^{255}S_{\mathrm{pillar}}\left(128,y\right)\right),$$
2. Maximum Attenuation ($A_{\max}$): The maximum signal drop compared to the no-pillar baseline.
  (4) $$A_{\max}=\underset{y\geq 128}{\max}\left[S_{\mathrm{base}}\left(128,y\right)-S_{\mathrm{pillar}}\left(128,y\right)\right],$$
3. Shadow Area ($Area_{\mathrm{shadow}}$): The integrated volume of the downstream signal attenuation.
  (5) $$Area_{\mathrm{shadow}}=\sum_{y=128}^{255}\max\left(0,\;S_{\mathrm{base}}\left(128,y\right)-S_{\mathrm{pillar}}\left(128,y\right)\right)$$

The analytical results are summarized in Table 2.

These quantitative data demonstrate the clear directional dependence of the flow shadow generated by the pillar. For the 12 o’clock frontal flow, a stark signal drop ($\Delta S_{\mathrm{diff}}\approx 0.030$) and large shadow area confirm strong localized gas dilution behind the pillar. For the 45° diagonal flow, a moderate intermediate attenuation ($\Delta S_{\mathrm{diff}}\approx 0.011$) was observed, consistent with the oblique interaction between the gas stream and the pillar. For the 9 o’clock lateral flow, which travels parallel to the wall-shaped pillar, the signal difference was near-zero ($\Delta S_{\mathrm{diff}}\approx 0.001$), indicating minimal shadow formation. By standardizing the evaluation axis, these findings clearly and quantitatively validate the robust angular selectivity of the proposed method.

### 4.2. Advantage over Conventional Methods and Positioning of the Proposed Method

Most conventional gas-source localization methods rely on the comparison of the concentration gradients generated by gas diffusion using multiple sensors. This approach is effective in calm environments; however, as noted above, in high-speed unidirectional flow (advection)-dominant environments, such as outdoors or within ventilation systems, turbulence destabilizes the concentration gradient, posing an inherent difficulty in direction estimation [32].

The proposed method offers a promising solution for this problem. Instead of relying on microscopic information like concentration gradients, it captures a clear and stable physical pattern—the “shadow”—forcibly generated by the structure, giving it the potential to function robustly even under high-speed gas flow. Indeed, a distinct “shadow” was successfully captured even within the high-speed flow conditions considered in this experiment.

Importantly, this method is unlikely to replace conventional techniques completely; rather, there is a high possibility of complementary relationships. For instance, a next-generation gas-sensing system capable of accommodating a wider range of environments, from calm conditions to strong winds, is expected to be realized by dynamically switching between the concentration gradient method and the proposed method based on flow speed, or by constructing a hybrid algorithm that integrates both [6].

As recent trends highlight the importance of system-level integration in intelligent sensing systems and embedded gas-monitoring architectures [33], conventional multi-sensor networks often face challenges related to architectural complexity. In contrast, our single-chip sensor offers significant advantages for integration into IoT devices and drones. Since this study focused on verifying the fundamental fluid-dynamic mechanism under idealized conditions, future practical deployments will need to address environmental variables such as wind fluctuations and humidity. The development of compensation algorithms incorporating temperature and humidity sensors constitutes a clear direction for our future work.

## 5. Conclusions

In this study, we proposed a novel method for estimating gas inflow direction by in-stalling pillar structures on a CMOS gas sensor array and observing the “shadow” generated by the gas flow, and we experimentally verified its basic principle.

Experimental results confirmed that a stable “shadow” clearly correlating with the gas inflow direction is formed on the downstream (wake) side of the structure. This finding suggests the potential of the proposed method for robust direction detection even in high-speed gas flow environments where conventional concentration-gradient methods tend to be unstable.

Based on these results, we developed a gas-direction detection sensor using a pillar. Future work will focus on optimizing the pillar geometry to maximize the performance of this method and developing quantitative algorithms to calculate the angle of arrival from the “shadow” features, aiming to establish a more practical “gas direction detection sensor.”

## Figures and Tables

**Table 1:** Qualitative and semi-quantitative structural comparison of the proposed method with conventional gas direction estimation techniques.

Method	Principle	Spatial Constraint & System Complexity	Required Sensors	Robustness in High-Speed Airflow	Reference
Concentration Gradient	Measures concentration difference between sensors	High (Requires spatial separation)	Multiple (≥2)	Low (Turbulence disrupts gradient)	[4,6,7]
Time-of-Arrival Difference	Measures detection time delay between sensors	High (Requires specific distance to detect delay)	Multiple (≥2)	Low to Medium (Depends on flow predictability)	[4,5]
Proposed Method	Detects local flow “shadow” generated by a surface pillar	Low (Single-chip array with passive structure)	Single array	High (Leverages wake formation under high-speed flow)	This work

**Table 2:** Quantitative analysis of the 1D flow shadow profiles under various inflow angles.

Direction	Upper Mean (y < 128)	Lower Mean (y≥128)	$\mathrm{Upper}\;\mathrm{vs}.\;\mathrm{Lower}\;\mathrm{Diff}\;(\Delta S_{\mathrm{diff}})$	$\mathrm{Max}\;\mathrm{Attenuation}\;(A_{\max})$	$\mathrm{Shadow}\;\mathrm{Area}\;(Area_{\mathrm{shadow}})$
12 o’clock	0.0531	0.0228	0.0303	0.0372	2.9367
45°	0.0569	0.0460	0.0109	0.0236	1.8858
9 o’clock	0.0637	0.0623	0.0014	0.0109	0.5253

## Data Availability

The original contributions presented in this study are included in the article. Further inquiries can be directed to the corresponding author.
